# Characterizing social and cognitive EEG-ERP through multiple kernel learning

**DOI:** 10.1016/j.heliyon.2023.e16927

**Published:** 2023-06-07

**Authors:** Daniel Nieto Mora, Stella Valencia, Natalia Trujillo, Jose David López, Juan David Martínez

**Affiliations:** a Máquinas Inteligentes y Reconocimiento de Patrones, Instituto Tecnológico Metropolitano ITM - Medellín, Colombia; b Grupo de Investigación Salud Mental, Facultad Nacional de Salud Pública, Universidad de Antioquia UDEA - Medellín, Colombia; c Grupo de Neurociencias de Antioquia, Facultad de Medicina, Universidad de Antioquia UDEA - Medellín, Colombia; d Engineering Faculty, Universidad de Antioquia UDEA - Medellín, Colombia; e Universidad EAFIT - Medellín, Colombia

**Keywords:** EEG-ERP, Multiple kernel learning, Social neuroscience, Cognitive neuroscience

## Abstract

EEG-ERP social-cognitive studies with healthy populations commonly fail to provide significant evidence due to low-quality data and the inherent similarity between groups. We propose a multiple kernel learning-based approach to enhance classification accuracy while keeping the traceability of the features (frequency bands or regions of interest) as a linear combination of kernels. These weights determine the relevance of each source of information, which is crucial for specialists. As a case study, we classify healthy ex-combatants of the Colombian armed conflict and civilians through a cognitive valence recognition task. Although previous works have shown accuracies below 80% with these groups, our proposal achieved an F1 score of 98%, revealing the most relevant bands and brain regions, which are the base for socio-cognitive trainings. With this methodology, we aim to contribute to standardizing EEG analyses and enhancing their statistics.

## 1 Introduction

For the last 20 years, the Colombian government has conducted peace agreements with illegal armed groups (guerrillas and paramilitaries) that have demobilized more than fifty thousand ex-combatants [1]. However, their return to civilian life has presented many obstacles due to high recidivism (going back to delinquency) and prejudice from civilians [2]. Previous studies have focused on identifying flaws in the government's reintegration programs. As a result, EEG-ERP studies have evidenced that the ex-combatants show an atypical emotional processing [3] that, after being identified, has helped improve social-cognitive trainings [4].

However, these studies have faced challenges in obtaining statistical significance. The populations under study are healthy, demographic differences among groups are subtle, the volunteers typically have low literacy, and the EEG must be recorded on site (in their villages, due to personal security concerns). These factors might hinder EEG analyses from capturing the main effects. For example, a Machine Learning (ML) and EEG functional connectivity study focused on finding differences between ex-combatants and civilians found significant differences in positive valence stimuli (Beta frequency band) [5]. However, results showed high intra-group variance and relatively low accuracy (below 80%). In a continuation study, the authors included cognitive behavioral tasks and achieved a classification accuracy of 85% [6]. A misclassification analysis found that many Colombian civilians have suffered from this long-term conflict as much as ex-combatants. Specifically, their neural response to ambiguous stimuli is similar to that observed in ex-combatants. This finding was confirmed with a novel psychological scale that measures exposure to extreme experiences [7]. Consequently, there is still a need to develop methods to identify electrophysiological differences between Colombian ex-combatants and civilians.

Several authors have faced a similar conundrum in their social-cognitive studies and used the common ML approach [8], [9]: (i) they extract features from raw EEG/MEG or ERP data, (ii) define the best features using a selection approach, and (iii) feed a classifier/regressor with the selected features. For example, [10] automatically classified responses to visual stimuli from a Cognitive Valence Recognition Task (CVRT) with IAPS images (International Affective Picture Reading) using genetic algorithms to select spatio-temporal features, followed by a Linear Discriminant Analysis (LDA) classifier. In [11], authors showed how ML allows identifying novel data-driven features in ERP analyses. They used ERP and a time-frequency approach to find inter-individual differences in a Go/Nogo task. Each feature was used individually to feed a Support Vector Machine (SVM) classifier. As a result, the accuracy achieved by each feature was used to assign its relevance to the studied task. [12] analyzed threat faces in infants (up to 3 years old) with an ERP task. The authors grouped EEG electrodes into several Regions-of-Interest (ROIs) to compute spatio-temporal features. Then, such features fed a Support Vector Regressor (SVR) to predict early-stage neural responses to threats measured with an eye-tracking strategy. Like the previous work, the relevance of each feature was measured from the parameters of the SVR. Finally, authors in [13] estimated the stop-signal reaction time from ERPs. They used an elastic net-like approach to determine the relevance of spatio-temporal features.

Furthermore, several other authors have used ML approaches to tackle EEG-related problems, mainly in clinical populations [14] or for finding emotional states (e.g., for brain-computer interfaces) [8], [15], [9]. For example, [16] designed a new set of features based on the Collatz Conjecture and applied the Iterative Neighborhood Component Analysis (INCA) to select clinically significant features to improve schizophrenia diagnosis. A similar approach was applied in [17] for Parkinson's disease detection, but features were computed using a graph-based aspirin model. For cognitive decline, [18] proposed an attention-based approach feed with features extracted from the EEG frequency bands (delta, theta, alpha, beta).

Although these methodologies achieve competitive performance metrics, social neuroscience specialists require interpretable features and outcomes. Particularly in our social-cognitive task, a classification methodology must help the psychologist to answer two essential questions: (i) which brain regions are informative? (ii) which frequency bands (rhythms) have predictive information? Regarding the first question, traditional feature selection methods do not consider the knowledge about brain functioning, i.e., the brain behaves as a comprised mosaic of patches with piece-wise constant functions that must be accounted for [19], [20]. From a machine learning perspective, features extracted from neighboring electrodes should contribute to the prediction process as a single group. For the second question, brain rhythms are highly correlated with cognition [21].

The abovementioned questions could be efficiently addressed by combining information from different sources, such as ROIs and frequency bands. One alternative is deep learning. In [22], authors fused features extracted from individual EEG channels using Convolutional Neural Networks (CNNs), reaching an outstanding accuracy for schizophrenia detection. However, this algorithm requires a significant amount of data unavailable for our study. As an alternative, Multiple-Kernel-Learning (MKL) algorithms were designed to combine feature groups within kernel-based machines. These techniques, successfully applied for various learning tasks (classification, regression, clustering), aim to optimally combine kernel matrices obtained from multiple sets of features with their corresponding weights [23], [24], [25], [26]. Even in recent years, MKL has taken advantage of the rapid advance of deep learning in what is known as deep kernel learning [27]. Close to our problem, MKL has been used to analyze the EEG signals from a cognitive task [28], but they used the kernels to represent a single feature set. Therefore, their weights inform about the best kernel but not the best feature, losing traceability.

Here, we propose a methodology to process EEG data from tasks or populations expected to present subtle (but existent) differences in leveraging information from different ROIs and frequency bands through an MKL machine. As a result, kernel weights inform about the main features that characterize each output (ex-combatant or civilian in our case). Our methodology includes a preprocessing pipeline, a typical time-frequency EEG characterization, and an MKL classifier as the main novelty of this work. We validate it by clustering a group of ex-combatants from illegal armed groups of the Colombian conflict and Colombian civilians with different levels of exposure to the conflict. The dataset consists of a CVRT based on IAPS and synchronized with EEG. Finally, the outcome of the MKL is compared with a traditional ML approach, and the results are analyzed from the neuropsychological perspective.

## 2 Materials and methods

### 2.1 Participants

The participants were 19 Colombian ex-combatants from the government's Agency for Reincorporation and Normalization program and 31 subjects with no history of participation in legal or illegal armed groups. Both groups were paired by gender, age, and educational level. Before the evaluation, all participants voluntarily read and signed an informed consent which contains the aim of the study and procedures description. The study had the ethical approval from the Faculty of Medicine of the University of Antioquia, Medellín, Colombia. A trained psychologist performed an initial short individual interview to discard psychiatric and neurological disorders that had required medical care.

### 2.2 Contextual valence recognition task

**Figure 1:**
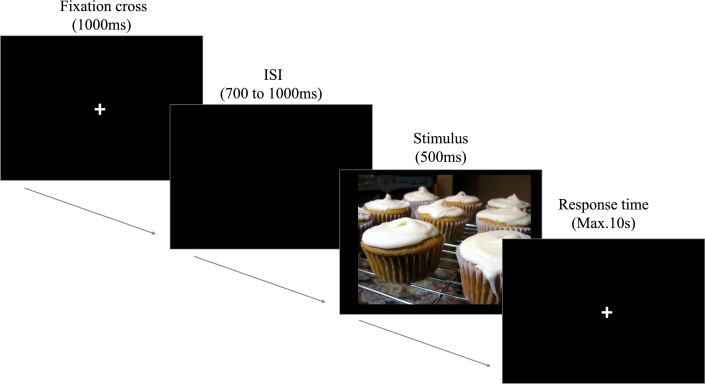
CVRT. The subject must respond if the displayed image (from an IAPS repository) is positive, neutral or negative.

EEG recordings from all subjects were acquired while performing a CVRT, which has been previously employed in ex-combatants to identify the valence of contextual images [5], [4]. The CVRT consists of presenting pictures from IAPS [29] categorized according to the valence reported in the Colombia validation by [30]. Sixty pictures were selected for three valence conditions: 20 positive, 20 neutral, and 20 negative. A total of 240 trials were presented, divided into four blocks of 60 trials each, and every image was presented once per block in a random order. The CVRT was implemented in E-prime (Psychology Software Tools, Pittsburgh, PA, United States) and presented in a 17-in PC screen at 60 cm from the participants. The sequence begins with a fixation cross during 1000 ms, followed by an inter-stimulus interval rating from 700 to 1000 ms, after which the stimuli were presented (positive, neutral, and negative IAPS pictures) for 500 ms. Finally, a participant response fixation-cross for a maximum of 10 s was set (see Fig. 1).

### 2.3 EEG acquisition and pre-processing

EEG recordings were acquired with a NeuroScan EEG SynAmps2 amplifier at a sample rate of 1 kHz. The 64 electrodes were placed according to the 10-20 international system. Impedances were maintained below 10 kΩ and the recordings were made in a Faraday cage with controlled lights.

All recordings were pre-processed using the same automatic pipeline (codes available under request). We carried out the following steps to attenuate and remove artifacts from environmental and biological sources: (i) Band-pass filtering, (ii) Re-referencing, (iii) Detection, removal, and interpolation of bad channels and bad epochs, (iv) Detection and removal of artifacts based on Independent Component Analysis (ICA). The preprocessing was performed using the MNE python package [31].

In the first step of the pre-processing, as we focused on event-related brain signals below 60 Hz, a band-pass filter from 0.1 to 60 Hz was applied to the raw EEG data. The high-pass filtering is intended to remove the DC drift and some body movements, whereas the low-pass attenuates the power-line interference of 60 Hz and remaining high frequency noise, avoiding the need for a notch filter. We used a zero-phase finite impulse response (FIR) filter to avoid affecting the evoked signal of interest. Once the data was band-pass filtered, it was re-referenced to an average in order to avoid diminishing the amplitude of data from electrodes near mastoids.

The next step consisted on extracting data epochs time-locked to the CVRT events described above. Thus, we defined trials of 1 s long including a -200–0 ms period that was used for baseline correction. With trials defined, we applied an automatic artifact rejection algorithm (*autoreject*) which learns peak-to-peak rejection thresholds using a data driven approach to correct or reject contaminated data segments [32]. This algorithm rejects an epoch only if a considerably large number of sensors agree that the trial is bad. In the opposite, few bad sensors for such trial are interpolated using spherical splines [33].

The last pre-processing step to complement *autoreject* is to detect and remove artifacts based on ICA. Common physiological artifacts as eye-blinks and heart beats have skewed and peaky distributions; therefore, they are easily captured by ICA methods that look for non-Gaussian sources [34]. As EOG channels were available, we used the Pearson correlations to find EOG related components. Moreover, ICA components corresponding to ECG activity were identified using cross-trial phase statistics (CTPS) [35]. As a result, for each *i*-th subject we obtained an EEG array $Y^{\left(i\right)}\in {ℜ}^{N_{c}\times N_{t}\times N_{e}}$, where $N_{c}$ is the number of channels, $N_{t}$ the number of time-samples, and $N_{e}$ the number of trials.

**Figure 2:**
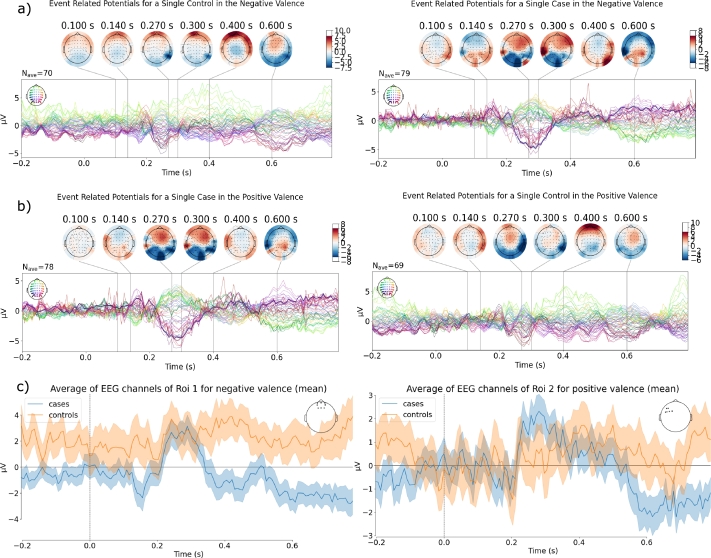
Event-related potentials from a single ex-combatant (case) and civilian (control). ROI 1 and ROI 2 were used to compare the mean of EEG signals for negative and positive valences, respectively. In a) ERPs for negative valence are shown, in b) ERPs for positive valence are shown, and in c) the average of EEG channels of ROI 1 was computed for negative valence, and the average of EEG channels of ROI 2 was computed for positive valence.

Once EEG data was pre-processed, the epochs belonging to each of the three valences described in section 2.1 were averaged together. As a result, for each *i*−th subject we obtained three evoked response potentials $Y^{\left(i\right)}\in {ℜ}^{N_{c}\times N_{t}}$ (one per valence). Fig. 2 shows examples of the obtained ERPs for negative valence (Fig. 2.a), positive valence (Fig. 2.b), and average of EEG channels for each ROI in negative and positive valences (Fig. 2.c).

### 2.4 Feature definition

**Figure 3:**
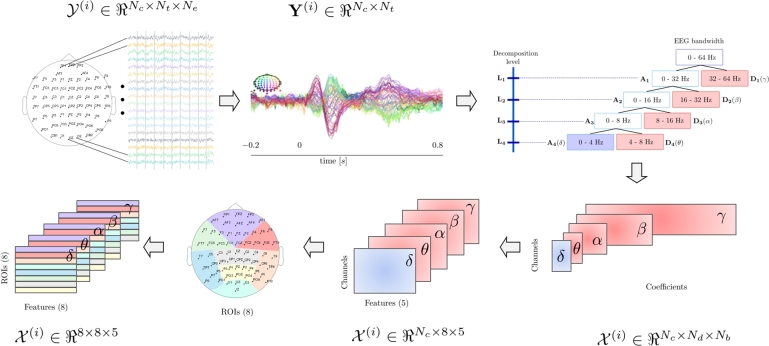
Feature extraction process: First, raw data is averaged over trials to extract the ERP. Using Wavelets, the ERP of each channel is filtered into five ERP rhythms. Then, some statistical features of each rhythm and each channel are computed. Finally, features belonging to the same ROI are averaged together to obtain the final feature set. The process is repeated for all subjects in the database.

We used a feature extraction approach based on the space-time-frequency information provided by the coefficients of the Discrete Wavelet Transform (DWT) of each channel. Consequently, the DWT coefficients in four decomposition levels were computed for each EEG channel using Symlet 8 sym4 as mother wavelet. We selected the detail coefficients $D_{1}$, $D_{2}$, $D_{3}$, and $D_{4}$, along with the approximation coefficients $A_{4}$ that approximately match with the $N_{b}=5$ frequency bands: *δ* (0–4 Hz), *θ* (4–8 Hz), *α* (8–16 Hz), *β* (16–32 Hz), and *γ* (32–60 Hz) (because of the low-pass filter applied as part of the pre-processing), as shown in Fig. 3.

As a result, for each EEG we obtained a coefficient array $X^{\left(i\right)}\in {ℜ}^{N_{c}\times N_{d}\times N_{b}}$, where $N_{b}=5$ denotes the number of decomposition levels (frequency bands), and $N_{d}$ stands for the number of coefficients in each decomposition level. We use the same notation $X^{\left(i\right)}$ through the entire characterization process for simplicity. Next, we computed the following statistics over the decomposition coefficients of each channel and frequency band: maximum value, minimum value, mean, median, variance, standard deviation, skewness, and kurtosis, obtaining a feature array $X^{\left(i\right)}\in {ℜ}^{N_{c}\times 5\times 5}$. To reduce the amount of features, we divided the EEG layout into eight Regions of Interest (ROIs), as shown in Fig. 3. Thus, all statistics from channels belonging to the same ROI were averaged together, giving as a result a feature array $X^{\left(i\right)}\in {ℜ}^{8\times 5\times 5}$. Finally, such array was flattened into a vector $x^{\left(i\right)}\in {ℜ}^{200}$ that comprises all the features obtained for subject *i*. As a result, we conducted several experiments with subsets of the 200 features to feed the classification methods described below.

### 2.5 Support vector machines and multiple kernel learning

#### 2.5.1 Single kernel SVMs

Once the features are computed, the next step is to train a classifier to separate among the considered groups: civilians (negative class) and ex-combatants (positive class). Despite the large amount of available classifiers, we chose SVMs for comparison purposes. It is a well-known classifier which uses the structural risk minimization theory to find a decision boundary that properly generalizes the prediction of unknown data in scenarios with a low ratio of sample size to dimension [36].

Briefly, SVMs are a kind of linear classifiers that learn a hyperplane maximizing the separating margin between positive and negative samples. Thus, given a set of training examples $x^{\left(i\right)}\in {ℜ}^{N_{f}\times 1}$ with i=1,…,N being *N* the number of subjects and $N_{f}$ the number of features, with their corresponding class labels $l^{\left(i\right)}\in \{-1,+1\}$, the primal SVM optimization problem is defined as:

(1) $$\min_{w,b,\xi }\;\frac{1}{2}||w|{|}_{2}^{2}+C\sum_{i=1}^{N}\xi_{i}\;\text{s.t.}\;y^{\left(i\right)}(w^{⊤}x^{\left(i\right)}+b)\geq 1-\xi_{i}\;\xi_{i}\geq 0,$$

where $w\in {ℜ}^{N_{f}}$ and b∈ℜ are the hyperplane parameters, $\xi_{i}\in {ℜ}^{+}$ are slack variables, and $C\in {ℜ}^{+}$ is the regularization parameter. By using Lagrange multipliers, the optimization problem in eq. (1) is transformed to its dual formulation as in eq. (2).

(2) $$\max_{\alpha_{i}}\;\sum_{i=1}^{N}\alpha_{i}-\frac{1}{2}\sum_{i=1}^{N}\sum_{j=1}^{N}\alpha_{i}\alpha_{j}l^{\left(i\right)}l^{\left(j\right)}k(x^{\left(i\right)},x^{\left(j\right)})\text{s.t.}\;\alpha_{i}\geq 0\sum_{i=1}^{N}\alpha_{i}y_{i}=0$$

where $k(x^{\left(i\right)},x^{\left(j\right)})=\phi {\left(x^{\left(i\right)}\right)}^{⊤}\phi (x^{\left(j\right)})\in {ℜ}^{+}$ denotes a kernel function. As a result, as the kernel might be seen as mapping the features to a high dimensional space, this formulation: (i) solves non-linear classification tasks through the kernel function and its implicit mapping ϕ(⋅), and (ii) deals with problems where only a small number of samples are available [37].

#### 2.5.2 Multiple kernel learning

One of the biggest challenges when using SVMs is to choose the suitable kernel and its parameters. This challenge worsens when features come from many sources, such as ROIs and frequency bands. Moreover, a single kernel is not enough to define the relevance of each feature, or at least each group of features. This constraint may significantly reduce the performance and interpretability of the SVM with challenging data (such as in our case). To avoid this issue, MKL combines kernels derived from multiple sources in a data-driven way to improve the accuracy of a kernel-based machine and provides a more flexible and practical framework to mine the information [38].

The most common approach to fuse kernels is through a linear combination:

(3) $$k_{\eta }(x^{\left(i\right)},x^{\left(j\right)})=\sum_{p=1}^{N_{p}}\eta_{p}k_{p}(x^{\left(i\right)},x^{\left(j\right)}),$$

where $N_{p}$ is the number of sources, $\eta =[\eta_{1},\ldots ,\eta_{N_{p}}]\in {ℜ}^{N_{p}\times 1}$ is a vector comprising the weights of each kernel, and $k_{p}(x^{\left(i\right)},x^{\left(j\right)})$ is a single kernel function calculated for a single information source with $\eta_{p}$ being its assigned weight. With certain constraints, the $\eta_{p}$ weights allow identifying the most relevant sources, i.e., features that provide insightful information to solve the classification task.

Here, we used the EasyMKL algorithm (eq. (4)) proposed in [39]. In short, imposing the constraint $\eta_{p}>0$, EasyMKL aims to solve the linear combination using a cost function that aims to maximize distance between positive and negative samples:

(4) $$\max_{||\eta |{|}_{2}=1}\min_{\xi \in \Xi }(1-\lambda )\xi^{⊤}L\left(\sum_{p=1}^{P}\eta_{p}K_{p}\right)L\xi +\lambda ||\xi |{|}_{2}^{2},$$

where $\lambda \in {ℜ}^{+}$ is a regularization parameter, $K_{p}\in {ℜ}^{N\times N}$ denotes the complete kernel matrix with evaluations of the *p*-th kernel for each pair of subjects, and $L\in {ℜ}^{N\times N}$ is a diagonal matrix where the *i*-th entry is the label of the *i*-th sample. Moreover, $\xi \in {ℜ}^{N\times 1}$ belongs to the domain of probability distributions Ξ defined over the set of positive and negative samples. The minimization over *ξ* provides the two nearest points in the convex hulls of positive and negative examples in the feature space induced by the kernel combination constraining the possible choices of **K**. The maximization over the unitary vector *η* aims to maximize the distance over the above-identified points. As a result, the larger the $\eta_{p}$ value, the more relevant the *p*-th information source is to solve the classification task.

### 2.6 Classification

One of the main drawbacks of using machine learning in cognitive tasks is its poor performance against challenging data (e.g., due to noise, few subjects, or not highly discriminative paradigms). This is the main reason for us to propose MKL. Because as indicated above, although the purpose of the classification stage is to better discriminate among classes (ex-combatants vs. controls), we expect traceability to determine which valences, ROIs and frequency bands are relevant for this classification. This additional information is critical for the specialist (e.g., neurologist or psychologist) to provide proper training/treatment.

For comparison purposes, we implemented a single kernel strategy (as a baseline for our validation) and the proposed MKL approach. First, the eight features corresponding to each ROI and the five frequency bands were used to train a single kernel SVM, yielding 8×5=40 classifiers. As a result, this experiment inspected the ROIs and frequency bands that presented more separation capability among the analyzed classes. We repeated the procedure for the three considered valences: positive, neutral, and negative. Then, we repeated the experiment by replacing the single kernel SVM with the proposed MKL, yielding 40 kernels and a single classifier per valence.

This first experiment discriminates valences due to their psychological importance, i.e., we are looking for a practical meaning of our findings. However, in practice, sometimes we only require to separate both groups (as in this scenario, ex-combatants vs. civilians). With this aim, we performed a second experiment with the same comparison but first collapsing valences.

### 2.7 Validation pipeline

Each experiment was validated with five-folds stratified cross-validation. In each fold, we used z-score normalization with respect to the mean and variance of the training set to take all features to the same scale. The Radial Basis Function (RBF) showed in eq. (5) was used as kernel:

(5) $$k(x^{\left(i\right)},x^{\left(j\right)})=\exp(\sigma ||x^{\left(i\right)}-x^{\left(j\right)}|{|}_{2}^{2}),$$

where $\sigma \in {ℜ}^{+}$ defines the influence ration of a single training example. For single kernel SVMs, this hyperparameter was set as $\sigma =1/(d\;\mathrm{var}(x_{\mathrm{train}}))$, where $\mathrm{var}(x_{\mathrm{train}})\in {ℜ}^{+}$ stands for the variance of all features in the training set; whereas for mulikernel SVMs, the hyperparameter was set as 1 because $\eta_{p}$ in eq. (3) should handle with the variations among feature spaces. We used 80% of the data for training, fixing the hyperparameter C=1 in eq. (1) to simplify the training process. Then, the label of each *j*−th sample of the remaining 20% was computed as in eq. (6):

(6) $$y^{\left(j\right)}=\mathrm{sign}\left(\sum_{i=1}^{N_{\mathrm{train}}}\alpha_{i}l^{i}k(x^{\left(i\right)},x^{\left(j\right)})+b\right),$$

where each $\alpha_{i}$ and *b* were learned in the training process, and $l^{i}$ is the label of the *i*−th training sample.

When using multiple kernels, the weights *η* of each kernel and the SVM parameters were learned in the training set as described in eq. (7). Then, the label of each *j*−th sample of the validation set was computed as:

(7) $$y^{\left(j\right)}=\mathrm{sign}\left(\sum_{i=1}^{N_{\mathrm{train}}}\alpha_{i}l^{i}\left(\sum_{p=1}^{P}\eta_{p}k_{p}(x^{\left(i\right)},x^{\left(j\right)})\right)+b\right).$$

We used F1 score as performance metric to account the class imbalance in our dataset. In brief, F1 score is the armonic mean of precision (number of true positives divided by the number of all samples identified as positives) and recall (number of true positives divided by the number of real positives). The reported performance metric is the average and standard deviation of the F1 score computed on the validation set for each fold.

## 3 Results

### 3.1 Single kernel vs. MKL separating valences

**Figure 4:**
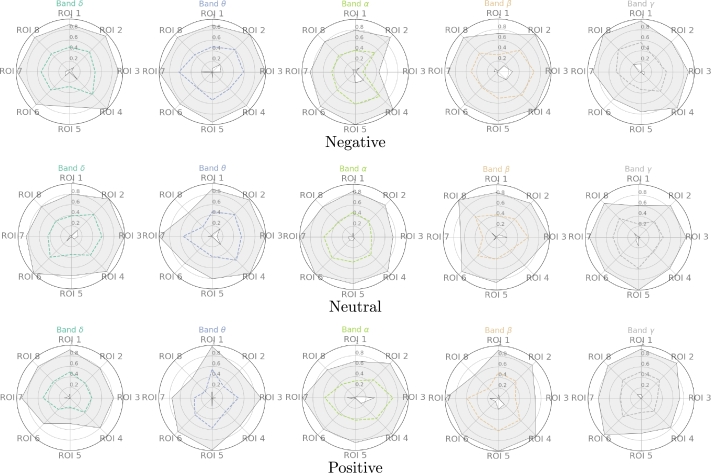
ML results. The low mean and large standard deviation evidence the impossibility for a single kernel to classify the groups.

Fig. 4 shows radar plots with F1 score mean (dashed line) and standard deviation (gray) for the single kernel approach at each considered valence. Classification for every ROI and frequency band shows a low mean and a high standard deviation, indicating a poor performance separating the two groups (ex-combatants and civilians). These results remain for all the considered valences. As a result, the single kernel approach cannot discriminate between classes. Moreover, the significant standard deviation indicates that for some subjects, either the classification is almost perfect (overfitting) or does not classify accurately any sample (underfitting). This result might be because the eight features computed for each ROI and frequency band cannot create a representation space where the analyzed groups are separable, making the classification boundary highly dependent on the SVM parameters.

**Figure 5:**
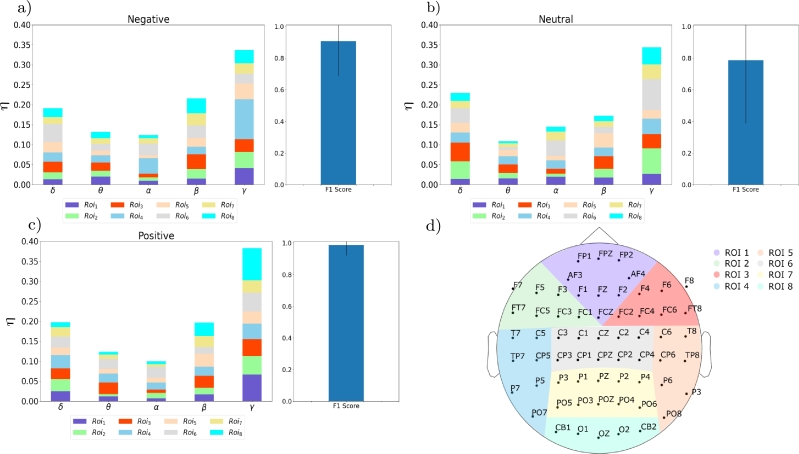
MKL results. Note how all valences reach significantly higher F1 scores than with the traditional SVM.

Fig. 5 shows the weights and F1 score (mean and standard deviation) achieved by the MKL approach. Regarding classification performance, the positive valence (Fig. 5.a) obtains the highest F1 score (up to F1 = 98%), followed by the negative (F1 = 90% Fig. 5.c) and neutral valences (F1 = 80% Fig. 5.b). The MKL approach overperforms the single kernel performance even in the less discriminative valence. This result indicates that the kernel combination eases the classification process of the SVM; i.e., the representation space to which the input features are mapped allows discriminating between the two considered groups. Moreover, we noticed that positive and negative valences are more discriminative than neutral. This result is expected because processing emotional stimuli (positive or negative) requires neural resources that differentiate from those needed for processing neutral stimuli and can help discriminate between the two groups [40]. In contrast, neutral stimuli might produce similar neural responses between controls and ex-combatants that do not allow discrimination.

Additionally, the weight distribution provides insight into the band and ROI discrimination relevance for the different valences. To facilitate comprehension, the color scheme of each bar directly mirrors the color designated to its corresponding ROI as shown in eq. (3). Note that these weights are not directly related to neural condition but to the differences between ex-combatants and civilians. I.e., a high weight value in *β*-band for ROI_8_ at the Positive valence means that there is a significant difference among groups and not that the CVRT is showing higher activity at the given region and frequency band. This result is helpful for the specialist, as the main objective in this kind of social-cognitive studies is to determine brain-behavior differences in a given group (in this case, ex-combatants) with respect to what is expected (e.g., civilians) for a given situation or condition (e.g., images with positive valence). Overall, *γ*-band is the most relevant; however, for negative stimuli, the relevance appears in temporal areas, whereas for positive stimuli, the most relevant information appears in the occipital brain area. For the positive and negative stimuli, we also noticed a high relevance in *β*-band over occipital areas, which have been related to attention and visual perception [41], [42].

### 3.2 Single kernel vs. MKL collapsing valences

**Figure 6:**
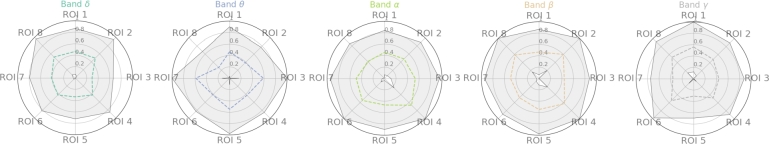
ML results. The low mean and large standard deviation evidence the impossibility for the single kernel to classify the groups, even with fewer features.

We then collapsed the valences together to feed the classifier with more information. Therefore, the eight features of each ROI and frequency band were concatenated through the valences (negative, neutral, and positive) to create a 24-feature vector. Results in Fig. 6 show that the single kernel approach does not improve when aggregating valences, indicating that regardless of the ROI and frequency band, a single set of features cannot provide helpful information to separate between controls and ex-combatants.

**Figure 7:**
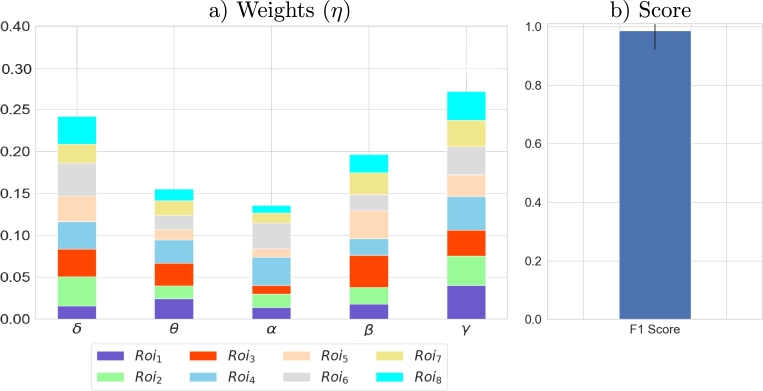
MKL results collapsing valences. The F1 score keeps the maximum level achieved with separated valences.

Moreover, Fig. 7 shows obtaining results of experiments collapsing valences and employing MKL to combine information from all the ROIs and frequency bands. Although the classifier can separate between groups (F1 score about 98% in Fig. 7.b), aggregating valence features does not provide any new insightful information, as the weight distribution (Fig. 7.a) looks precisely like the one obtained for the positive valence. Furthermore, for the sake of interpretability, it is more helpful for the psychological team to analyze valences independently; for this reason, this experiment is valuable solely from the machine learning point of view.

## 4 Discusion

This study aimed to develop a methodology for processing EEG data from social-cognitive tasks, wherein subtle differences are expected and traditional methods struggle to achieve significant results. Our work's novelty lies in the incorporation of an MKL classifier designed to amplify differences between groups while maintaining input-output traceability. We tested the classifier by distinguishing between two challenging groups: Colombian ex-combatants and civilians. The MKL classifier demonstrated satisfactory classification, with the Delta, Beta, and particularly Gamma bands showing higher relevance across various ROIs. We will discuss the proposed methodology and its potential implications in the following sections.

### 4.1 Proposed methodology

Numerous approaches have been proposed for preprocessing EEG recordings to enhance classification tasks. Preprocessing is a critical step, as it significantly influences the quality and reliability of the extracted information. For simplicity, we employed an automated methodology comprising three steps: enhancing signal quality through pass-band filters, extracting time-locked data epochs, and removing artifacts using the autoreject technique and ICA. The obtained ERPs (see Fig. 2) indicate that the preprocessing effectively emphasizes patterns that aid in distinguishing between the studied groups.

In the subsequent step, we extracted features using DWT. This analysis offers several advantages over other characterization techniques, such as its ability to localize and describe time-frequency patterns aimed at emphasizing group differences; its capacity to decompose signals into multiple levels, enabling the description of ERPs in frequency bands that are highly interpretable for neuropsychologists; and its robustness to noise, which reduces the dependence of feature extraction on preprocessing steps. Moreover, we chose to average the extracted statistical features computed for each decomposition level in ROIs to decrease the feature space dimensionality, thus facilitating the classifier's performance while providing additional interpretability.

Once the features were computed, the next step involved training a classifier to differentiate between the control and ex-combatant groups. A variety of algorithms are available, including linear models, neural networks, tree-based models, and ensembles. We opted for a multi-kernel approach due to its capability to combine features from diverse sources. Typically, MKL is employed when features originate from distinct acquisition processes (e.g., images and signals). In our study, we utilized MKL to merge information from various frequency bands and ROIs. The results demonstrated that this approach not only enhances classification performance but also generates information that can be easily analyzed by the neuropsychologist team, thereby increasing the proposed method's utility. Of all the available MKL algorithms, we selected easy-MKL for its user-friendliness and the direct correlation between the obtained weights and the kernels' relevance.

### 4.2 Implications of the achieved results

Human emotion generation and affective processing are complex and sensitive to subjective aspects such as attachment patterns [43], [44], [45]. Additionally, in social neuroscience the differences among groups are subtle [43]. ML approaches have been extensively used to analyze EEG signals from features extracted with help of expert knowledge about the underlying neural process [8], [9]. But their accuracy when facing social cognitive tasks in healthy populations is still low [6]. Moreover, they fail to keep traceability of sources such as frequency bands, which have been associated with mental processes such as attention, learning, working memory, spatial navigation, and emotional recognition [46], [42], [47], [48].

Here, we tackled these traceability and accuracy issues with an MKL-based processing pipeline. The MKL approach aims to linearly combine kernels computed from different sources of information to create a representation space where the subjects of both groups are linearly separable. In this sense, MKL can discard features that do not contribute to the groups' separability through the linear combination of weights and highlight relevant features. As a result, such weights provide insights that the specialist can interpret as potential biomarkers of the social-cognitive task.

Regarding the results achieved in the case study, the proposed MKL approach overperforms the traditional single kernel SVM classifier in all the tested scenarios. Consequently, we confirmed experimentally that a proper combination of information on spatial and frequency features enhances differences between groups.

The results of our study show that the Gamma band over prefrontal, central, temporal, and occipital regions is relevant to classify Colombian Ex-combatants and civilians in the three emotional valences evaluated (positive, neutral, and negative). In this regard, the literature has described Gamma and Beta bands as relevant when classifying emotions as valence and arousal [49]. Furthermore, other studies have reported for the Gamma band that, for stimuli with negative or positive valences, a higher response in prefrontal and temporal areas, respectively, are expected [50]. Accordingly, our findings show a significant difference between the studied groups for the negative valence over prefrontal regions (ROI_1_) and positive valence over temporal ones (ROI_2_). This result reveals a difference in neural patterns of Colombian ex-combatants over civilians during emotional processing as a potential adaptive response to war contexts. Confrontations and armed conflicts require a series of skills necessary to process the information surrounding war contexts to read possible threats and to act quickly when necessary, especially in stressful situations. In this sense, exposure to war can lead to adaptive mechanisms (i.e. behavioral or emotional regulation) in order to survive in violent contexts.

The Beta band has also been identified as a helpful frequency band for classifying emotional recognition. Similar to the Gamma band, we found that the relevance of the Beta band over temporal areas is higher for the positive valence in comparison with neutral and negative stimuli [51], [50]. Studies in ex-combatants report significant differences in the Beta band when processing emotional information from civilians over central-parietal, and lateral parietal-occipital regions [9]. Our results show differences in the Beta band for positive and negative valences over both temporal and occipital areas. The Beta band over occipital areas has been related to attention and visual perception processes [41], [42]. This implies that the beta-band may contextually modulate or allocate attentional resources to classify the input information. This process is especially relevant for those who are in threatening situations.

Finally, we found that the Delta band is also relevant to classify the groups in the positive valence over almost all ROIs except occipital areas. Moreover, for the neutral valence, this band is relevant over lateral, temporal, and central areas, while for the negative valence, it is mainly relevant over central areas. During mental tasks, Delta band is associated with cognitive processes such as attention, concretely attending to a stimulus while inhibiting distractors [47], [48]. Our results follow those of [47], where they observed that Delta band functional connectivity was reorganized in ex-combatants after a social cognition training, suggesting that the Delta band is effectively reorganized in ex-combatants and is subject to intervention by the specialists.

In summary, our findings provide a characterization with an MKL-based classifier that identified Delta, Beta, and Gamma bands as potential markers to classify different emotional valences among ex-combatants and civilians, both healthy populations from similar demographics. The findings suggest, in consonance with previous studies from our research, that experience related to armed conflict drives a neural reorganization potentially with survival proposes. Thus, the ex-combatants' emotional processing supports a faster and more accurate identification of contextual threats, consequently triggering a response.

The proposed approach has two main limitations: (i) it is susceptible to the used kernel and its parameters, and (ii) the combination of MKL and SVM that we propose can only be used when dealing with two groups. The first can be easily solved by including such parameters in the cross-validation technique or by using MKL to combine the same kernel with different parameters. For example, in [28], authors proposed a methodology where they computed different kernels with the same features and then combined such kernels with an MKL approach to analyze the EEG signals from cognitive tasks. The second limitation is harder to solve, as both the cost function for computing the weights of the linear combination of kernels and the classifier should be modified. In future works, we expect to use different sets of features with neurological interpretability, such as functional connectivity. Then, this approach will provide insights into the best possible characterization for processing cognitive tasks based on EEG.

## 5 Conclusions

In this paper, we proposed a novel EEG processing and classification approach based on MKL. The proposed approach creates a feature representation space by combining features from different information sources, such as ROIs (space) and frequency bands, that allows separating between the studied groups and identifying the most relevant sets of features. We tested our approach by finding neural spatial and frequency variations between ex-combatants from the Colombian armed conflict and Colombian civilians during a CVRT. One of the most important findings in this study is how multi-kernel learning maintains, on average, good class separability, even achieving higher discrimination than the best band used to train separately support vector machines. This indicates that MKL performs an effective combination of information and, in some cases, generates better decision boundaries in the dataset mapped to the feature plane. The input/output traceability allowed us to identify the principal ROIs for negative and positive contextual valences. We concluded that ROI_1_, composed of frontal sensors, was associated with negative valence changes, and left frontotemporal sensors were related to changes in the positive valence. Additionally, we managed to identify Delta, Beta, and Gamma bands as potential biomarkers to classify different emotional valences. These results are consistent with the literature and, with higher accuracy than previous works (achieving 98% F1-Score, which considers precision and recall), provide a better insight into the problem to the specialist. With this methodology, including the preprocessing pipeline, we expect to contribute to standardizing cognitive and social neuroscience EEG analyses.

## Ethics statement

This article builds upon data collected under project MinCiencias 495-2020, and Colciencias number FP44842-525-2014. Research procedures were approved by Universidad del Rosario's Research Ethics Committee (Minute DVO005-063-CS048, February 8, 2018). All participants voluntarily read and signed an informed consent which contains the aim of the study and procedures description.

## CRediT authorship contribution statement

**Daniel Alexis Nieto-Mora, Juan David Martínez-Vargaz, Stella Valencia, Natalia Trujillo, Jose Lopez:** Conceived and designed the experiments; Performed the experiments; Analyzed and interpreted the data; Contributed reagents, materials, analysis tools or data; Wrote the paper.

## Declaration of Competing Interest

The authors declare that they have no known competing financial interests or personal relationships that could have appeared to influence the work reported in this paper.

## Data Availability

Data will be made available on request.
